# Our initial experience with rapid enzymatic debriding agent for burn eschar: Case series from an ABA verified burn center

**DOI:** 10.1016/j.burnso.2025.100403

**Published:** 2025-03-29

**Authors:** Cole L. Bird, Yair Saucedo, Jessica Reynolds, Dhaval Bhavsar

**Affiliations:** a University of Kansas, School of Medicine, Kansas City, KS, USA; b MidAmerica Nazarene University, Department of Natural, Health, and Mathematical Sciences, Olathe, KS, USA; c Department, University of Kansas, Department of Plastic Surgery, Kansas City, KS, USA

**Keywords:** NexoBrid^®^ (anacaulase-bcdb), Enzymatic debriding agent, Burn, Bromelain

## Abstract

We reviewed 14 consecutive patients at our ABA-verified burn center who received enzymatic debridement with anacaulase-bcdb (NexoBrid^®^) from January 2020 to May 2023. These patients, part of the NEXT study, had deep partial or full-thickness burns. We aimed to evaluate NexoBrid’s effect on eschar removal, wound healing, surgical needs, and scar quality.

Data included total body surface area (TBSA) burned, enzymatically treated area, amount of NexoBrid used, grafting details, time to healing, and scar characteristics. Analysis was descriptive, reporting medians, ranges, and percentages.

All 14 patients achieved ≥ 95 % eschar removal with a single NexoBrid application. Their ages ranged from 15–65 years, and mean burn size was 9.25 % TBSA. Eight patients required grafting, but these grafts covered only about 60 % of the treated area. Time to 95 % wound closure averaged 36 days. Scar assessment using the Vancouver Scar Scale showed improvement from a mean score of 3.8 at three months to 0.5 at twelve months. Despite this, four patients developed hypertrophic scars and one required intervention for a contracture.

In summary, NexoBrid facilitated rapid, consistent non-surgical eschar removal, timely wound closure, and favorable scar outcomes within one year. In nearly half of the patients, it eliminated the need for skin grafting. Among those who did require grafts, smaller graft areas were needed. These findings suggest that early eschar removal and dermal preservation contribute to improved outcomes. Further studies with larger cohorts will help confirm these results.

## 1. Introduction

Burn wound excision is a critical component of managing patients with deeper burn injuries. Approximately 5 % to 20 % of burn patients require surgical intervention for optimal recovery [1]. According to the latest data from the National Burn Registry, any surgical approach to burn treatment begins with surgical wound debridement [2]. Traditional methods of debridement, including tangential and fascial excision, aim to promote healing by removing necrotic tissue [3]. Specifically, tangential excision involves the careful removal of necrotic tissue to create an optimal wound bed. To achieve this, it is imperative to eliminate all burn eschar completely. However, current surgical techniques are less precise and often result in removal of viable dermis, which can impact the quality of the resulting scar [4].

The FDA recently approved a novel rapid enzymatic debriding agent, NexoBrid^®^ (anacaulase-bcdb) [5]. This agent facilitates rapid, non-surgical eschar removal, typically achieved in a single four-hour application. Emerging international studies highlight the promising outcomes associated with NexoBrid, suggesting its efficacy in enhancing wound healing and improving scar quality [6]. However, there is a notable paucity of research conducted within the United States focusing on these critical aspects of healing and scar quality following its use.

In this context, we aim to retrospectively review our experience with NexoBrid as part of the NexoBrid Expanded Access Protocol (NEXT) study (MW2018–06–21). Our objective is to analyze the enrolled patients regarding eschar debridement, wound healing, need for surgical wound closure and scar quality outcomes.

## 2. Methods

Eligible patients were adults (≥18 years old) with thermal burns involving more than 1 % total body surface area (TBSA) of deep partial-thickness or full-thickness injury requiring eschar removal. Patients with a total burn extent of less than 30 % TBSA were included, and all provided informed consent within 84 h of injury. Burn depth was determined by clinical assessment from an attending burn surgeon. Fifteen patients were initially screened and consented for NexoBrid use; however, one patient withdrew consent prior to application. Data from the remaining 14 patients were de-identified and recorded in an Excel-based data collection tool for analysis.

From there we began analysis, focusing on six key factors (Table 1).

### 2.1. Nexobrid application

Patients with deep partial and full thickness burn wounds were selected for the study. Patients were presented with the study information and consent. Enrolled patients were then planned for application of enzymatic debriding agent for deep partial and full thickness burn wounds. Target wounds were covered with antibacterial solution soaked gauze for at least 2 hrs (≤12 hrs). Vaseline ointment barrier was created around the edge of the target wound over the healthy skin. Enzyme was applied to the wounds per manufacturer’s guidance and then sealed with a plastic wrap followed by bulky dressing. Enzymatic debridement was continued for 4 hrs. At the end of that duration, wounds were cleaned of all materials and scraped of loose debrided eschar. Wounds soaked again for 2 hrs and were evaluated for completion of eschar removal (≥95 % eschar). After the completion of enzymatic debridement, wounds were dressed and skin grafting procedure was planned as needed.

## 3. Results

**Fig. 1.**
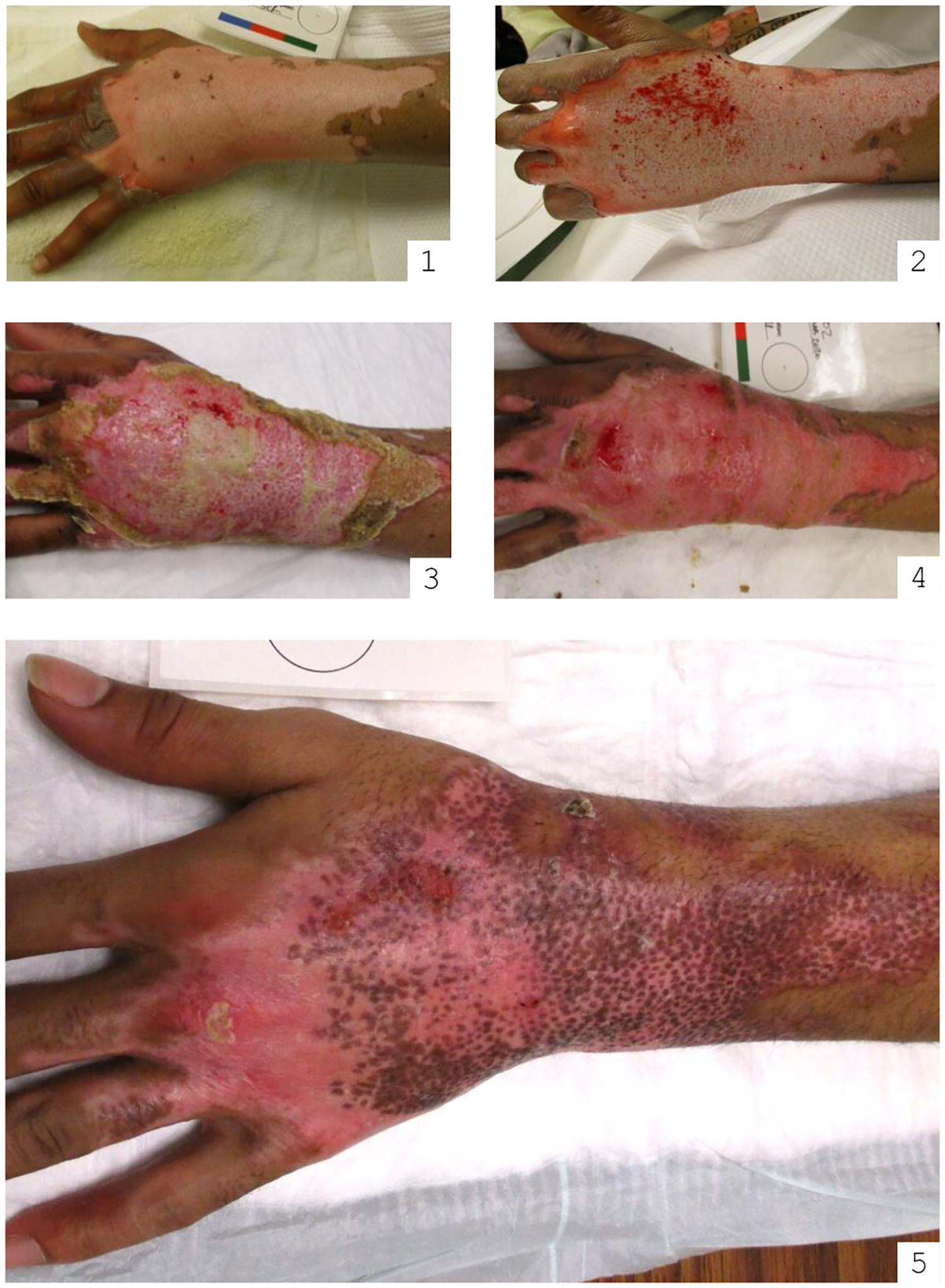
Progression of deep partial thickness/full thickness anterior chest burn following treatment of Anacaulase. Patient photo series. (1) – pre-treatment (post burn day 1); (2) – immediately post-treatment; (3) – 2 weeks post; (4) – 3 weeks post; (5) – 1 month post.

Fourteen patients treated with NexoBrid were included in the final analysis. The cohort was predominantly male (79 %), with an age range from 14 to 68 years and an average age of 36 years. The average TBSA affected by burns was 9.25 % ± 7 % (see Table 2 for a summary of patient demographics and treatment outcomes). The average amount of NexoBrid applied per patient was 12.3 g. Skin grafts were required in 57 % of cases, with the area grafted averaging 63 % ± 31.1 % of the enzymatically treated burn wound TBSA. An example of the clinical progression is seen in Fig. 1.

Opioid usage for post-operative pain management was standardized to morphine milligram equivalence (MME). Common opioids included IV hydromorphone, IV fentanyl-citrate, and oral oxycodone. The average MME totals for the first three post-operative days were 58.8, 26.0, and 18.3, respectively. Notably, 71 % of participants received intra-operative ketamine drips.

The average time to achieve 95 % wound closure was 36 days (± 11.06 days). Vancouver Scar Scale scores improved significantly over the study period, decreasing from an average of 3.8 ± 2.8 at the 3-month follow-up to 0.5 ± 1.3 at one year (*p*-value = 0.00373). Four patients (28.6 %) developed hypertrophic scars, and one patient (7.1 %) developed a contracture.

## 4. Discussion

This study provides our early experience with the use of NexoBrid for rapid enzymatic debridement of burn eschar. It highlights the agent’s effectiveness in achieving complete eschar debridement, our primary goal. Nexobrid was successful in complete removal (≥95 %) of burn escahr in all 14 patients. The rapid and efficient enzymatic debridement observed in this cohort aligns with the existing literature, which suggests that NexoBrid can achieve removal of necrotic tissue efficiently similar to the traditional surgical methods [6]. The ability to achieve greater precision in debridement potentially reduces the risk of inadvertently removing viable dermis, which could improve scar quality and long-term cosmetic outcomes [4].

A notable finding from our study is that 43 % of patients treated with NexoBrid did not require skin grafting, despite the presence of at least deep partial-thickness burn injury. Among those who did require grafting, the average grafted area represented only 63 % of the total burn wound treated with NexoBrid. Since we chose the deep partial and full-thickness burns which otherwise would have been routinely grafted in our practice, the observation that the total area ultimately grafted was smaller than the total area initially treated suggests that NexoBrid may have contributed to dermal preservation. This may be due to NexoBrid facilitating more precise eschar removal, leading to dermal preservation and potentially preserving the epidermal appendages. While this study did not include a direct comparison group, this observation supports the potential of NexoBrid in reducing the need for grafting. The reduced need for grafting could be especially beneficial in high-risk patients, where minimizing the extent of surgical intervention is critical for improving outcomes and reducing complications. This finding warrants further investigation in larger cohorts with controlled comparisons to confirm the role of NexoBrid in minimizing the need for grafting and its broader implications for burn care.

The timing of wound healing in this cohort was also promising. The average time to reach 95 % wound closure was 36 days, which is within the expected range for enzymatic debridement and comparable to prior reports. While enzymatic debridement has been associated with earlier wound bed preparation and potential benefits for re-epithelialization, healing trajectories can vary based on treatment pathways. Importantly, this study followed an intent-to-treat (ITT) model, meaning all enrolled patients—including those who opted out of grafting—were included in the analysis. Since grafting typically expedites wound closure, the inclusion of these non-grafted patients likely prolonged the reported healing time. However, the fact that these patients achieved wound closure without surgical intervention further supports the potential role of enzymatic debridement in optimizing the wound environment and facilitating healing. This suggests that NexoBrid may support wound healing in some cases without additional surgical intervention, potentially reducing the overall burden on the patient.

Scar quality, as assessed by the Vancouver Scar Scale (VSS), was highly encouraging during the study period, with mean VSS scores of 3.8 ± 2.8 at three months and 0.5 ± 1.3 at one year (p = 0.00373). These findings align with the promising outcomes of NexoBrid in previous studies, which reported improved scar outcomes with enzymatic debridement, likely due to more controlled and effective eschar removal [7,8]. Notably, NexoBrid has demonstrated reduced scar hypertrophy and improved pliability compared to conventional methods in large cohorts of patients with full-thickness burns. Published data from large studies show that hypertrophic scars occur in approximately 26 %–35 % of patients with full-thickness burns, with the incidence of contractures ranging from 3 % to 9 % [9,10]. Our incidence of hypertrophic scarring was 28.6 %, and the incidence of contracture was 7.1 %. While our cohort is smaller, these rates align closely with those seen in larger studies, which report an incidence of 26 %–35 % for hypertrophic scarring and 3 %–9% for contractures in comparable burn populations.

Regarding scar quality, our observed 3.3-point reduction in the VSS score from baseline to one-year post-injury is notable. Published studies have shown reductions of 2.8–4.2 points on the VSS for patients treated with advanced therapies such as enzymatic debridement and laser therapy, with larger cohorts consistently reporting similar improvements [7,10]. This suggests that NexoBrid is likely comparable to other leading treatments for scar improvement.

Despite these positive outcomes, there are limitations to our study that must be addressed. First, the retrospective nature of this analysis limits our ability to control potential confounders. Additionally, the sample size of 14 patients is small, and further studies with larger cohorts are needed to confirm these findings. The lack of a comparison group is another limitation, as it is unclear whether these outcomes are significantly better than those achieved with traditional burn excision methods. Future randomized controlled trials would help to elucidate the comparative effectiveness of NexoBrid against other treatment modalities.

Another factor to consider is the variable response to pain management. Although the standardization of opioid use in terms of morphine milligram equivalence (MME) helped to track pain management across patients, the use of intra-operative ketamine drips in 71 % of participants may have influenced postoperative analgesia and recovery times. Further studies investigating the impact of adjunctive analgesia on post-operative outcomes could be valuable.

In conclusion, NexoBrid appears to be a valuable tool for the non-surgical removal of burn eschar, with potential benefits in terms of precise debridement, reduced need for skin grafting, and improved scar quality. Given the promising results in this cohort, further research with larger, controlled studies is warranted to confirm its effectiveness and determine the ideal patient population for its use. In particular, its potential application in high-risk surgical patients, where minimizing surgical intervention is critical, warrants further exploration.

## 5. Conclusion

Our retrospective review of 14 patients treated with NexoBrid for eschar removal demonstrates its viability as an alternative to traditional surgical debridement, achieving complete eschar removal in all cases. Notably, 43 % of patients did not require grafting, and among those who did, the grafted area averaged only 63 % of the treated burn wound. This suggests that NexoBrid offers more precise debridement, potentially reducing the need for extensive grafting. The rapid eschar removal also facilitates earlier and more accurate wound depth assessment. Most patients achieved 95 % closure within an average of 36 days, and scar quality improved significantly over time. While skin grafting was required in 57 % of cases, our results indicate that NexoBrid is a promising tool for burn wound management, particularly for high-risk surgical patients. Future studies should explore the long-term outcomes of NexoBrid, including its impact on quality of life and functional recovery, and the economic impact of integrating NexoBrid into clinical practice.

## Figures and Tables

**Table 1:** Six key factors.

Key Factors	Description
**TBSA Treated**	Date of NexoBrid Application Target Wound Depth Skin Graft Requirement % of TBSA Treated
**Time to Heal (Days)**	Date of official wound closure Additional complications extending healing times
**Skin Graft**	Details of skin graft harvesting Application and take
**Pain Management Strategy**	Timing Type of medication Strength of medication Duration of medication
**Scar Assessment (VSS)**	Color (vascularization and pigmentation) Thickness (height)Relief (surface irregularities)Pliability (tissue elasticity)Surface Area (scar contraction or expansion)
**Adverse Events**	Type of event Severity of event
**Follow Up/Further Intervention**	If secondary treatment required

**Table 2:** Summation of results.

Variable	NexoBrid^®^ (anacaulase-bcdb) Averages
**Gender**	11 Male (79 %)
	3 Female (21 %)
**Age (years)**	36 [range 14–68]
**Total Body Surface Area of Burn (TBSA)**	9.25 % ± 7 %
**Grams applied of NexoBrid^®^ (anacaulase-bcdb)**	12.3 g ± 8.1
**Use of Skin Graft**	57 %
**Percent TBSA of Treated Burn Grafted**	63 % ± 31.1 %
**Morphine Milligram Equivalence (MME)**	
Post-op Day 1	58.8 ± 32.8
Post-op Day 2	26.0 ± 21.1
Post-op Day 3	18.3 ± 17.3
**Time to heal (95 % wound closure)**	36 days ± 11.06
**Vancouver Scar Scale – 3 Month**	3.8 ± 2.8
**Vancouver Scar Scale – 1 year**	0.5 ± 1.3
